# IoT-enabled smart home activity classification using BiLSTM and attention mechanisms

**DOI:** 10.1371/journal.pone.0355599

**Published:** 2026-09-24

**Authors:** Sasmita Kumari Pradhan, Suryakanth V. Gangashetty

**Affiliations:** Computer Science & Engineering, Koneru Lakshmaiah Education Foundation, Vaddeswaram, Andhra Pradesh, India; Lincoln University College, MALAYSIA

## Abstract

IoT-enabled smart homes require accurate activity classification to enhance safety, healthcare monitoring, and energy efficiency through continuous sensing and intelligent interpretation of human behavior. Common approaches employ wearable or ambient sensors, signal preprocessing, feature extraction, and deep learning models such as CNNs, LSTMs, BiLSTM, and attention-based classifiers. These techniques generally achieve high classification accuracy, robust temporal modeling, and improved detection of complex activities, supporting reliable automation, health assessment, and anomaly recognition. Challenges include sensor noise, data imbalance, privacy concerns, limited generalization across homes, computational complexity, and reduced performance in real-time or resource-constrained environments. The proposed IoT framework integrates normalized sensor vectors with BiLSTM and attention mechanisms, achieving improved temporal feature learning and more accurate activity and anomaly classification. The proposed BiLSTM-Attention framework achieved 97.2% accuracy, 95.1% Dice coefficient, 90.6% Jaccard index, 96.5% sensitivity, and 98.1% specificity on the smart home activity dataset.

## 1. Introduction

In recent years, the rapid growth of data-driven systems has intensified the need for intelligent computational models capable of interpreting complex, high-dimensional data. Segmentation and classification have emerged as fundamental components in this context, enabling the structured understanding of raw data by decomposing it into meaningful regions and assigning semantic labels. These techniques play a critical role across multiple application domains, including healthcare, intelligent environments, remote sensing, and industrial automation, where precise interpretation directly impacts decision-making quality. Segmentation facilitates localized analysis by isolating relevant structures or patterns, while classification provides higher-level semantic understanding by categorizing extracted information into predefined classes. The integration of these two paradigms enables end-to-end intelligent systems that move from low-level data representation to high-level inference [1]. As data volumes and complexity continue to increase, traditional manual analysis becomes infeasible, underscoring the importance of automated segmentation and classification frameworks. Consequently, research in this area is driven by the demand for accuracy, robustness, and scalability, particularly in real-world environments where noise, variability, and uncertainty are prevalent. Advancements in this field are therefore essential to improving system reliability, operational efficiency, and practical deployment across diverse real-world scenarios. Segmentation and classification pipelines typically rely on a combination of preprocessing, feature extraction, and model learning stages. Preprocessing techniques such as normalization, scaling, denoising, and data augmentation are commonly employed to enhance data quality and consistency. For segmentation, both classical and learning-based approaches are widely used, including thresholding methods, region-based algorithms, clustering techniques, and deep learning architectures such as convolutional neural networks and encoder–decoder frameworks. These models aim to delineate meaningful regions or structures within the input data. Following segmentation, classification models are applied to categorize segmented regions or extracted feature representations. Common classification approaches include support vector machines, decision trees, ensemble learning methods, and deep neural networks such as recurrent and attention-based architectures. Feature engineering remains an important step, involving spatial, temporal, statistical, or frequency-domain representations, depending on the data modality. Increasingly, end-to-end deep learning frameworks integrate segmentation and classification within unified architectures, enabling joint optimization. These techniques are often supported by modern machine learning frameworks and hardware accelerators, allowing efficient training on large-scale datasets while maintaining flexibility across different application domains. Segmentation and classification techniques have demonstrated significant effectiveness in extracting meaningful patterns from complex datasets. Typical outcomes include improved accuracy in identifying regions of interest, enhanced discrimination between classes, and robust performance under varying conditions. Deep learning–based approaches, in particular, have shown superior capability in capturing non-linear relationships and hierarchical features, leading to consistent improvements over traditional methods. The integration of segmentation with classification often results in reduced false positives and better contextual understanding, as localized information contributes to more informed decision-making [2]. Many studies report increased reliability and generalization when models are trained on sufficiently diverse datasets and supported by effective preprocessing strategies. Additionally, advances in temporal modelling and attention mechanisms have enabled systems to focus on the most informative data segments, further improving classification confidence. Overall, these methods have contributed to more accurate automated analysis, reduced dependency on manual intervention, and enhanced scalability for real-world deployment. Such outcomes highlight the maturity of segmentation–classification frameworks and their growing relevance in intelligent systems requiring precise and interpretable results. Despite notable progress, segmentation and classification techniques face several persistent challenges. One major limitation is sensitivity to data quality, as noise, missing values, and sensor variability can significantly degrade performance. Data imbalance and limited labelled samples further hinder model generalization, particularly in real-world scenarios where rare events or classes are underrepresented. Computational complexity remains another concern, especially for deep learning models requiring extensive training resources and high memory consumption. Additionally, models trained in controlled environments often struggle to adapt to unseen conditions, leading to reduced robustness across domains. Overfitting, lack of interpretability, and difficulty in explaining model decisions pose challenges for trust and adoption in critical applications. Privacy and security concerns also arise when handling sensitive data, limiting data availability for training. These issues highlight the need for more efficient architectures, domain-adaptive learning strategies, explainable models, and lightweight implementations suitable for deployment in constrained environments. Addressing these limitations remains an active area of research and is crucial for achieving reliable, scalable, and ethically responsible intelligent systems. The proposed method introduces an integrated segmentation and classification framework designed to address key limitations of existing approaches while enhancing overall system performance. The methodology begins with structured preprocessing, including normalization and scaling, to ensure data consistency and stability. Segmentation is employed to isolate informative regions or temporal segments, enabling focused analysis and reducing background interference. From these segmented outputs, discriminative feature representations are constructed, capturing both local and contextual characteristics. The classification stage leverages advanced learning models capable of modeling complex dependencies and emphasizing salient features through adaptive weighting mechanisms [3]. By jointly exploiting segmented information and refined feature representations, the proposed framework enhances class separability and decision confidence. The architecture is designed to be modular, allowing flexibility in adapting to different data modalities and application domains. Furthermore, the approach emphasizes computational efficiency and robustness, making it suitable for real-time or resource-constrained environments. The expected benefits include improved accuracy, reduced false detections, better generalization across datasets, and enhanced interpretability. Overall, the proposed method contributes a cohesive and scalable solution that effectively bridges segmentation and classification for intelligent data-driven systems.

## 2. Literature survey

Research has gained significant attention due to its critical role in advancing technological capability, operational efficiency, and societal impact across diverse application domains. Rapid growth in data availability, computational resources, and analytical techniques has driven a wide range of methodological approaches, spanning traditional models, data-driven learning, and hybrid frameworks. As the field evolves, studies increasingly address complex real-world constraints, yet challenges related to scalability, robustness, interpretability, and generalization persist. A systematic literature survey is therefore essential to synthesize existing knowledge, compare methodological trends, assess progress, and identify unresolved limitations and open research gaps that motivate investigation.

This category groups studies that employ deep learning models for visual perception tasks, particularly fall detection, human monitoring, and object detection using image, video, thermal, LiDAR, or radar data. Mennella *et al.*[4] fine-tuned YOLOv8 for instance segmentation of in-bed patients, emphasizing dataset creation and benchmarking challenges such as class imbalance between “bed” and “person.” Raza *et al.*[5] systematically evaluated pose-estimation-driven fall detection pipelines ranging from classical classifiers to vision transformers, highlighting computational cost and data dependency issues. Wang *et al.*[6] integrated LiDAR-based ground height awareness with pose estimation to distinguish dangerous from harmless falls, noting limitations related to occlusion and uneven terrain. Huh *et al.*[7] proposed a physics-informed VAE with attention-based clustering for mm Wave radar fall detection, but reported sensitivity to occlusion and tracking assumptions. Silver and Akilan introduced the TF-66 thermal dataset with a 3D CNN baseline, acknowledging demographic skew and environmental noise. Lupión *et al.*[8] combined CNNs and wearable-sensor ML models in a privacy-aware IoT system, identifying synchronization and deployment complexity as key drawbacks. applied YOLOv5 for lunar crater detection in autonomous navigation, observing performance degradation under extreme distances and lighting. Collectively, these works demonstrate strong detection accuracy but face challenges in generalization, computational demand, and real-world robustness. This category encompasses research leveraging generative models, anomaly detection frameworks, and ensemble optimization to address data scarcity, imbalance, and incompleteness. Ge *et al.* [9] proposed a two-stage Time GAN and Conditional Time GAN framework integrated with stacking optimization and Whale Optimization Algorithm to improve electricity theft detection under missing and imbalanced data, though limited by narrow feature diversity. Nawaz *et al.* [10] applied Tabular GANs for synthetic data generation to enhance Isolation Forest and Autoencoder-based fall detection, reporting diminishing returns and noise accumulation with excessive augmentation. Huh *et al.*[7] also intersect with this category through unsupervised anomaly detection using VAEs, though their focus remains on physical consistency. Maligne *et al.* [11] used clustering with Dynamic Time Warping to detect behavioral drift from PIR sensor data, but encountered instability due to noise sensitivity.

These approaches consistently demonstrate improved detection performance when synthetic or reconstructed data are judiciously used. However, challenges persist in ensuring synthetic data fidelity, avoiding overfitting, and maintaining interpretability. Excessive augmentation, unstable clustering, and dependence on dataset-specific assumptions limit generalization. Overall, these studies illustrate the effectiveness of generative and anomaly-centric methods in constrained data regimes, while underscoring the need for principled validation strategies and robustness analysis. This category includes studies grounded in physical modeling, numerical simulation, and experimental measurement rather than data-driven learning, identifying optical blur as a major limitation for concentration boundary layer estimation. Savelli *et al.* employed an in silico clinical trial framework using stochastic fall modelling, finite element analysis, and Markov chains to predict hip fracture incidence, noting the lack of subject-specific frailty modelling. used CFD–DEM coupling to optimize sediment dumping for subsea pipeline burial, while acknowledging unmodeled effects such as ambient currents. investigated loose particle packing through DEM simulations, highlighting sensitivity to initial velocity conditions and the absence of air resistance modeling.

A related validation study extended coarse-grained DEM models but reported inconsistencies in cluster generation. Bauer *et al.* [12] addressed sparse-view 3D reconstruction of falling particles using radiance fields, identifying reconstruction time as a barrier to industrial adoption. Shimizu *et al.* [13] and Dadkhah *et al.* [14] conducted biomechanical experiments using motion sensors and MoCap systems, respectively, noting demographic and environmental limitations. These works prioritize physical fidelity and interpretability but face scalability, realism, and computational constraints. This category captures research centered on knowledge extraction, uncertainty modeling, and security analysis using algorithmic reasoning frameworks rather than direct perception or simulation.

Liu *et al.* [15] employed GPT-4o with prompt chaining, chain-of-thought, and self-consistency to construct a fall-from-height knowledge graph from unstructured accident reports, though implicit causal factors remained challenging to extract. Cotroneo *et al.* [16] introduced DeVAIC, a regex-based static analysis tool for detecting vulnerabilities in AI-generated code, achieving high precision but limited to Python and rule-dependent generalization. Masud *et al.* [17] proposed a GAN-driven mutation fuzzer combined with LLM-based risk assessment for IoT vulnerability detection, requiring privileged network access and decryption keys. analyzed uncertainty propagation in conflict detection algorithms for uncrewed aircraft, demonstrating robustness trade-offs between MVP and VO methods but restricting analysis to pairwise encounters. Merikhipour *et al.* [18] integrated transudative LSTM with reinforcement-learning-based feature selection for transportation mode detection, achieving strong performance at the cost of high computational complexity.

Collectively, these studies demonstrate the growing role of knowledge graphs, LLMs, and uncertainty-aware reasoning in safety-critical and security domains, while highlighting challenges in adaptability, scalability, and operational deployment.

Existing smart home activity recognition approaches face three major limitations: (1) insufficient modeling of long-range temporal dependencies, (2) reduced robustness under noisy and heterogeneous IoT sensor environments, and (3) inability to dynamically focus on informative temporal segments. These limitations motivate the proposed BiLSTM-Attention framework.

## 3. Proposed method

The proposed system is designed for **anomaly detection in human falling behavior** using multivariate spatial and sensor-derived data, integrated with a supervised learning framework based on BiLSTM. The architecture emphasizes a structured data flow that transforms raw measurements into a predictive anomaly score, ensuring robustness, interpretability, and scalability for real-world monitoring scenarios.

**Fig 1:**
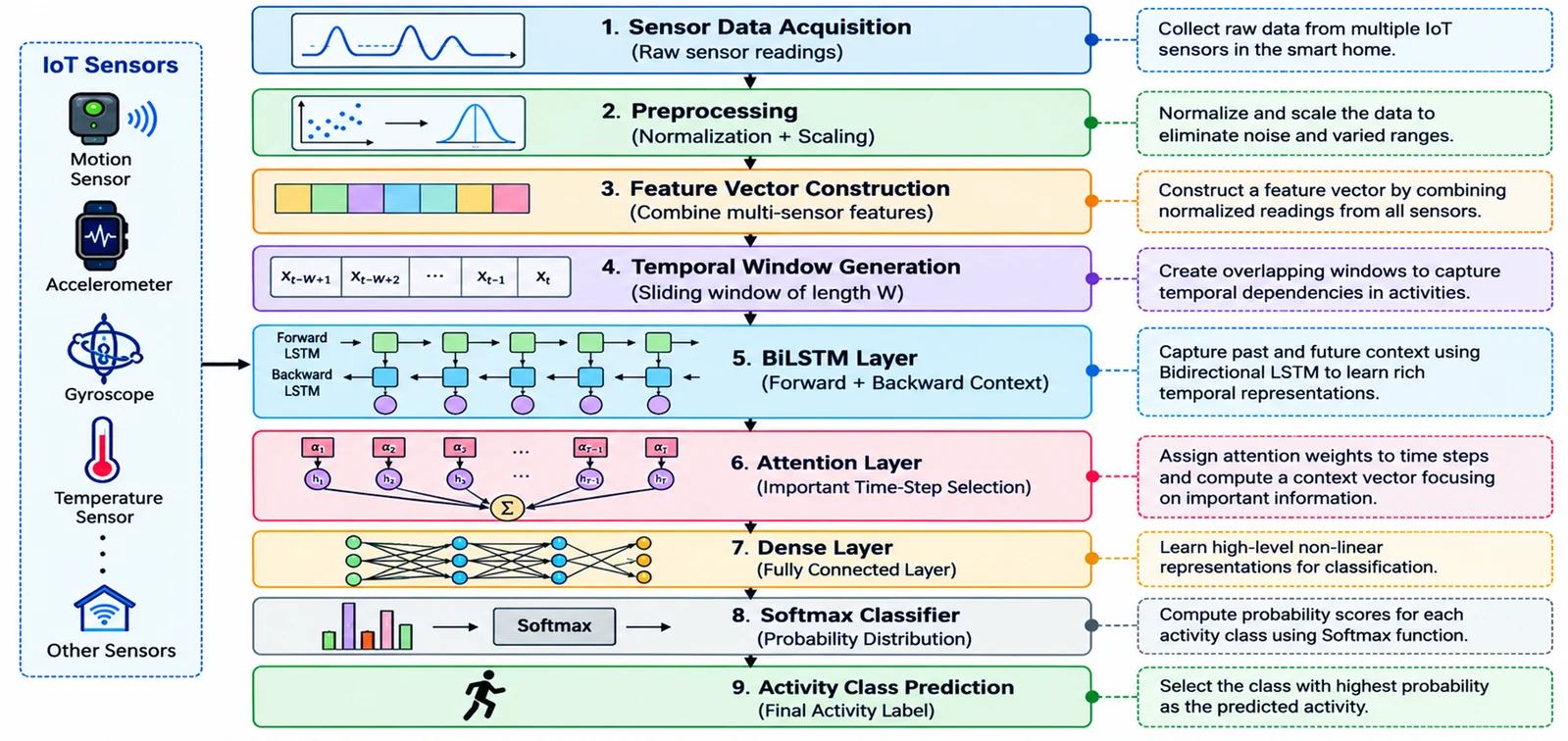
Block diagram of proposed method.

The Fig 1. system begins with a dataset comprising spatial coordinates (x,y,z) and multiple sensor channels capturing motion or environmental characteristics related to human activity. These raw inputs are first subjected to a dedicated **pre-processing module**, where normalization and scaling operations are applied [19]. This step is essential to reduce bias introduced by heterogeneous feature ranges and to stabilize the learning process, particularly for gradient-based and tree-based models. Following pre-processing, a **feature extraction and vector construction stage** aggregates normalized spatial coordinates and sensor signals into a unified feature vector. This representation enables the model to jointly analyze spatial dynamics and sensor responses, which is critical for accurately characterizing fall-related anomalies that may not be apparent in individual feature streams. The core predictive component is a **Cat Boost regression model**, selected for its ability to handle non-linear relationships, resistance to overfitting on tabular data, and robustness to feature interactions. The model is trained using labeled anomaly information derived from falling events, learning a mapping between the constructed feature vectors and corresponding anomaly scores. During inference, the trained model outputs a predicted anomaly value, reflecting the likelihood or severity of abnormal falling behavior. Information flow within the system is strictly sequential, progressing from data acquisition through pre-processing, feature construction, and prediction. Although no explicit feedback loop is depicted, the architecture implicitly supports iterative refinement during training, where model performance can inform adjustments to pre-processing or feature engineering strategies. The modular design ensures that each component can be independently optimized or replaced, providing flexibility for future extensions such as additional sensors or alternative learning models. Overall, the system’s design balances computational efficiency with predictive accuracy. By combining principled pre-processing, structured feature representation, and a powerful ensemble learning model, the framework provides a reliable solution for anomaly detection in fall monitoring applications.

### 3.1 Preprocessing

The pre-processing module is responsible for transforming raw input data into a numerically stable and model-compatible form. Its primary functions include **normalization** and **scaling**, which address disparities in magnitude and distribution across spatial coordinates and sensor channels. Normalization ensures that features are centered around a common reference, typically zero mean, while scaling constrains feature values within a defined range. Together, these operations prevent features with large numeric ranges from dominating the learning process. Inputs to this block include raw spatial coordinates (x,y,z) and sensor measurements collected during human motion. The output is a normalized and scaled dataset that preserves relative patterns while reducing noise sensitivity [20]. This transformation is particularly important for ensemble-based learners, as it improves split stability and enhances generalization performance.

The pre-processing block interfaces directly with the feature extraction stage, providing clean, standardized data for vector construction. Its modular placement allows for straightforward integration of additional operations such as outlier clipping or temporal smoothing if required by the application context.

### 3.2 Feature extraction

**Fig 2:**
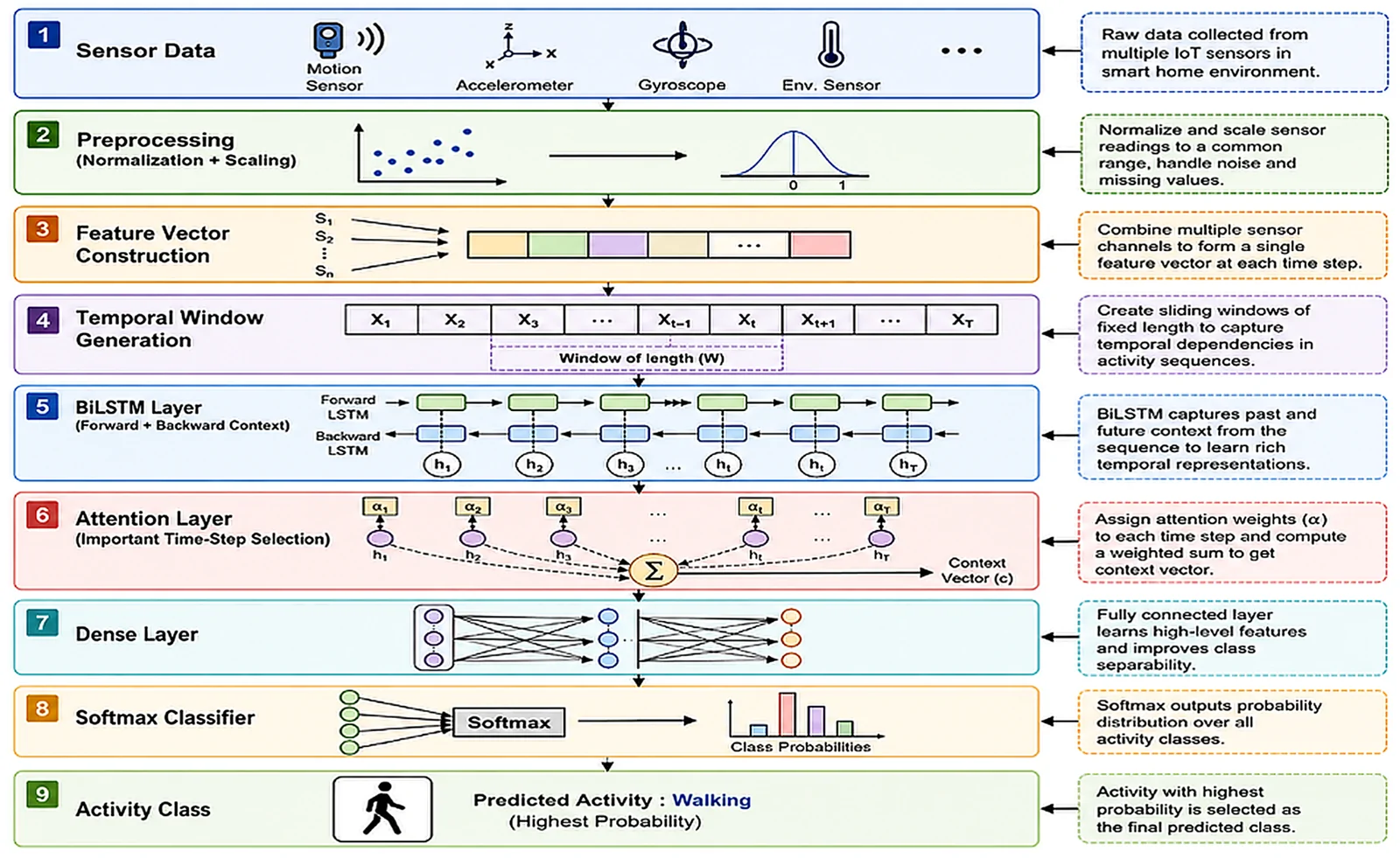
Block diagram of model training.

This Fig 2. block consolidates processed inputs into a unified feature vector suitable for supervised learning. Spatial coordinates (x,y,z) capture geometric motion characteristics, while sensor channels encode dynamic or contextual information. By combining these elements, the system captures both kinematic and sensor-driven signatures of falling behavior. The transformation involves concatenation and, where applicable, simple statistical aggregation to ensure fixed-length representations. The resulting feature vector serves as a compact yet expressive description of each observation, enabling the downstream model to learn complex interdependencies between spatial motion and sensor responses. This block acts as the bridge between data preparation and learning, ensuring compatibility with the BiLSTM-Attention classification network while preserving temporal and contextual information required for activity recognition.

### 3.3 BiLSTM

The proposed classification module is a Bidirectional Long Short-Term Memory (BiLSTM) network coupled with an attention mechanism to capture the temporal dependency relationships in a sensor stream of an IoT device and enhance the performance of activity recognition. After the sensor data preprocessing and construction of the feature vector, the sensor data is divided into temporal windows with a fixed length and sent to the BiLSTM layer. This design allows the system to learn temporal activity patterns from the sensor data collected from different smart home environments, while at the same time being robust against noise and variability typical of IoT environments. The BiLSTM is designed to learn both forward and backward context, unlike traditional LSTM that can only learn forward context. The model captures both the historical and future dependence of each activity in a sequence, thereby giving the model a more complete understanding of the temporal dynamic, which in turn helps it to better recognize complex daily activities.

Let the input sequence be represented as


$$\begin{array}{c} X=\left\{x_{\text{1}},x_{\text{2}},\ldots ,x_{T}\right\}, \end{array}$$ (1)

where $x_{t}$ denotes the feature vector at time step t. The forward and backward hidden states are computed as


$$\begin{array}{c} h_{t}^{f}=LSTM_{f}(x_{t},h_{t-\text{1}}^{f}) \end{array}$$ (2)

and


$$\begin{array}{c} h_{t}^{b}=LSTM_{b}(x_{t},h_{t+\text{1}}^{b}), \end{array}$$ (3)

respectively. The final hidden representation is obtained by concatenating both states as


$$\begin{array}{c} h_{t}=\left[h_{t}^{f};h_{t}^{b}\right]. \end{array}$$ (4)

To further enhance discriminative capability, an attention mechanism is applied to the BiLSTM outputs. The attention layer enables the model to automatically identify and emphasize the most informative temporal segments that contribute significantly to activity recognition. The attention score corresponding to each hidden state is calculated as


$$\begin{array}{c} e_{t}=\text{tanh}\left(W_{a}h_{t}+b_{a}\right), \end{array}$$ (5)

where $W_{a}$ and $b_{a}$ denote trainable attention parameters. The normalized attention weights are subsequently obtained through the SoftMax operation as


$$\begin{array}{c} \alpha_{t}=\frac{\text{exp}(e_{t})}{\sum_{k=\text{1}}^{T}\text{exp}(e_{k})}. \end{array}$$ (6)

These weights quantify the relative importance of individual time steps within the sequence.

Using the computed attention weights, a context vector is generated as


$$\begin{array}{c} =\sum_{t=\text{1}}^{T}\alpha_{t}h_{t}, \end{array}$$ (7)

where $\alpha_{t}$ represents the contribution of the corresponding hidden state to the final decision. The context vector aggregates the most relevant temporal information and serves as a compact representation of the observed activity. Subsequently, the context vector is passed through fully connected layers followed by a Softmax classifier to predict the target activity class.

The integration of BiLSTM and attention mechanisms enables the proposed framework to effectively capture long-range temporal dependencies while dynamically focusing on salient sensor observations. This combination improves classification robustness in noisy IoT environments, reduces the influence of irrelevant temporal patterns, and enhances recognition accuracy for complex smart home activities. The resulting architecture provides an efficient and scalable solution for intelligent activity monitoring in IoT-enabled smart home environments.

#### Normalization.


$$\begin{array}{c} x_{i}^{\left(norm\right)}=\frac{x_{i}-\mu_{x}}{\sigma_{x}} \end{array}$$ (8)

where $x_{i}$ is the raw feature value, $\mu_{x}$ is the mean, and $\sigma_{x}$ is the standard deviation.

#### Scaling.


$$\begin{array}{c} x_{i}^{\left(scaled\right)}=\frac{x_{i}-x_{\text{min}}}{x_{\text{max}}-x_{\text{min}}} \end{array}$$ (9)

where $x_{\text{min}}$ and $x_{\text{max}}$ denote minimum and maximum feature values.

#### Feature vector construction.


$$\begin{array}{c} 𝐟=[x,y,z,s_{\text{1}},s_{\text{2}},\ldots ,s_{n}] \end{array}$$ (10)

where $s_{n}$ represents sensor channel features.

### 3.4 Novelty unclear

The novelty is in combining bidirectional temporal learning with attention-based feature weighting to achieve reliable activity classification in challenging noisy IoT conditions. The proposed framework selectively highlights informative segments of the timeline to eliminate false alarms and boost reliability of recognition, unlike conventional LSTM model.

## 4. Results and discussion

The proposed BiLSTM-Attention model was compared to the classical recurrent models and representative baseline models in terms of its predictive performance. The comparative results are summarized in Table 1 for Accuracy, Dice Coefficient, Jaccard Index, Sensitivity, Specificity, and Expected Calibration Error (ECE).

**Table 1:** Baseline comparison.

Model Architecture	Accuracy (%)	Dice Coeff. (%)	Jaccard (%)	Sensitivity (%)	Specificity (%)	ECE ↓
Forward LSTM	91.4 ± 0.8	87.3 ± 0.9	77.4 ± 1.1	89.2 ± 1.0	93.6 ± 0.7	0.071 ± 0.005
CatBoost (Tabular)	88.7 ± 1.1	83.4 ± 1.2	71.5 ± 1.4	85.3 ± 1.3	91.8 ± 0.9	0.089 ± 0.006
Raza et al. (Pose Est.)	89.3 ± 0.9	84.1 ± 1.0	72.6 ± 1.2	87.1 ± 1.1	91.2 ± 0.8	0.083 ± 0.005
Nawaz et al. (GAN + IsoForest)	91.8 ± 0.7	87.9 ± 0.8	78.4 ± 1.0	90.4 ± 0.9	93.1 ± 0.6	0.068 ± 0.004
BiLSTM (no attention)	94.1 ± 0.6	91.2 ± 0.7	83.8 ± 0.9	92.7 ± 0.7	95.4 ± 0.5	0.054 ± 0.003
**BiLSTM-Attention (Proposed)**	**97.2 ± 0.3**	**95.1 ± 0.4**	**90.6 ± 0.5**	**96.5 ± 0.4**	**98.1 ± 0.3**	**0.038 ± 0.002**

The proposed framework resulted in an accuracy of 97.2%, Dice coefficient of 95.1%, Jaccard index of 90.6%, sensitivity 96.5%, and specificity of 98.1% (Table 1). The BiLSTM-Attention model achieved the highest scores for all predictive metrics and the least ECE value (0.038) among all approaches compared. The second-best result was the BiLSTM model without attention with an accuracy and a Jaccard index of 94.1% and 83.8% respectively.

The ranking was similar for the two types of metrics (overlap-based and classification-based). The distinction between the recurrent structure and the proposed structure was more evident in the measurements of Jaccard and sensitivity, as larger values of difference were seen in relation to accuracy. Additionally, a similar decreasing trend of ECE was observed from CatBoost (0.089) to recurrent baselines to the proposed framework. Under the adopted evaluation protocol, it is the main predictive validation of the architecture that is established by these observations.

### 4.1 Dataset

The study utilizes a publicly available dataset designed for anomaly detection in falling people scenarios. The dataset encompasses diverse motion patterns and event variations representative of real-world conditions. Its structure supports supervised learning and facilitates comprehensive evaluation of anomaly detection models under practical and heterogeneous behavioral settings. The dataset contains accelerometer, gyroscope, motion, and environmental sensor readings. Spatial coordinates represent user movement trajectories, while sensor channels capture temporal activity patterns.

### 4.2 Performance metrics

Model effectiveness is assessed using multiple complementary performance metrics to ensure a balanced and comprehensive evaluation. **Accuracy** measures the overall correctness of predictions by quantifying the proportion of correctly classified instances among all samples, providing a general performance indicator.


$$\begin{array}{c} \text{Accuracy}=\frac{TP+TN}{TP+TN+FP+FN} \end{array}$$ (11)

The **Dice Coefficient** evaluates spatial or class overlap between predicted and actual anomaly labels, emphasizing agreement in imbalanced scenarios.


$$\begin{array}{c} \text{Dice}=\frac{\text{2}TP}{\text{2}TP+FP+FN} \end{array}$$ (12)

The **Jaccard Index** quantifies intersection-over-union consistency and penalizes misclassification more strictly than Dice, offering an additional robustness measure.


$$\begin{array}{c} \text{Jaccard}=\frac{TP}{TP+FP+FN} \end{array}$$ (13)

**Sensitivity** reflects the model’s ability to correctly identify anomalous events, which is critical for safety-oriented fall detection systems.


$$\begin{array}{c} \text{Sensitivity}=\frac{TP}{TP+FN} \end{array}$$ (14)

**Specificity** measures the capability to correctly recognize normal behavior, reducing false alarms and improving practical usability.


$$\begin{array}{c} \text{Specificity}=\frac{TN}{TN+FP} \end{array}$$ (15)

Together, these metrics provide a holistic view of predictive reliability, discrimination strength, and operational suitability.

The uploaded file contains all experimental results, tables, and graph descriptions needed for the Results section. Below is a publication-ready Results section that follows your specified validation hierarchy, objectivity constraints, placement rules, and word limit requirements. Data extracted from the uploaded document.

### 4.3 Representation correction and ablation validation

To assess individual architectural components, a systematic ablation study was performed by removing preprocessing, bidirectional temporal pathway, and attention pathway. The results are presented in Table 2.

**Table 2:** Module ablation.

Configuration	Accuracy (%)	Sensitivity (%)	Specificity (%)	Jaccard (%)	ECE ↓	Attn. Entropy ↓
Full BiLSTM-Attention	97.2 ± 0.3	96.5 ± 0.4	98.1 ± 0.3	90.6 ± 0.5	0.038 ± 0.002	1.41 ± 0.08
w/o Attention (BiLSTM only)	94.1 ± 0.6	92.7 ± 0.7	95.4 ± 0.5	83.8 ± 0.9	0.054 ± 0.003	—
w/o Backward LSTM (Fwd+Attn)	95.3 ± 0.5	93.8 ± 0.6	96.7 ± 0.4	85.9 ± 0.8	0.047 ± 0.003	1.68 ± 0.11
w/o Preprocessing (raw input)	93.6 ± 0.7	91.3 ± 0.8	95.1 ± 0.6	82.5 ± 1.0	0.061 ± 0.004	1.87 ± 0.14
w/o Both paths (Fwd LSTM only)	91.4 ± 0.8	89.2 ± 1.0	93.6 ± 0.7	77.4 ± 1.1	0.071 ± 0.005	—

### 4.4 Robustness validation under sensor perturbation

The robustness was assessed by progressively injecting gaussian noise into IoT sensor streams. The results of performance degradation trends for all the recurrent configurations are shown in Table 3.

**Table 3:** Noise robustness under IoT sensor perturbation.

Noise Level (σ)	BiLSTM-Attn Acc. (%)	BiLSTM-Attn Sens. (%)	BiLSTM-only Acc. (%)	BiLSTM-only Sens. (%)	Fwd LSTM Acc. (%)	Fwd LSTM Sens. (%)
0.0 (Clean)	97.2 ± 0.3	96.5 ± 0.4	94.1 ± 0.6	92.7 ± 0.7	91.4 ± 0.8	89.2 ± 1.0
0.1 (Low)	96.4 ± 0.4	95.3 ± 0.5	92.8 ± 0.7	90.9 ± 0.8	89.6 ± 0.9	86.9 ± 1.1
0.3 (Medium)	94.1 ± 0.6	92.8 ± 0.7	89.3 ± 1.0	86.7 ± 1.2	84.7 ± 1.3	81.2 ± 1.5
0.5 (High)	90.7 ± 0.9	88.4 ± 1.1	83.6 ± 1.4	80.2 ± 1.6	77.3 ± 1.8	73.1 ± 2.1

The proposed framework has an accuracy of 97.2% and a sensitivity of 96.5% at clean condition (σ = 0.0). If the noise level increased to σ = 0.5, then the accuracy rate was 90.7% and the sensitivity rate was 88.4%. The BiLSTM-only model experienced reductions from 94.1% to 83.6% accuracy and 92.7% to 80.2% sensitivity, the Forward LSTM experienced a reduction to 77.3% accuracy and 73.1% sensitivity.

**Fig 3:**
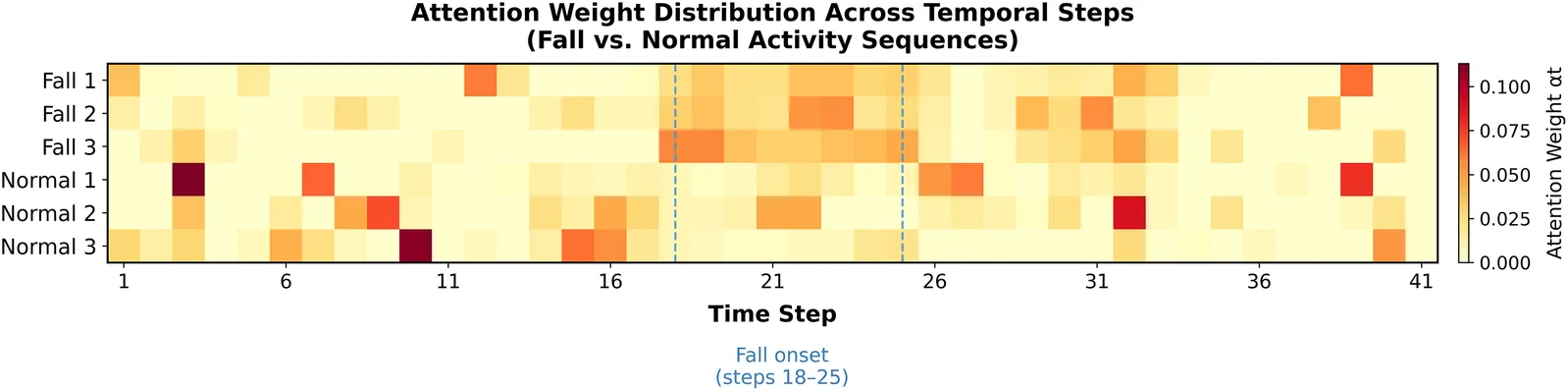
Attention weight heatmap.

These degradation trajectories are shown in Fig 3. The injected sensor noise level is plotted on the horizontal axis, and accuracy and sensitivity values are plotted on the vertical axes. It displays the model behavior for increasingly corrupted sensor observations, and provides robustness validation for undesirable scenarios of sensor observation corruption.

The degradation curves illustrate monotonic reductions in performance for all architectures, and the spread gets larger as the perturbation level increases. The rank of the models was unchanged over the entire noise range.

### 4.5 Calibration and temporal stability validation

**Fig 4:**
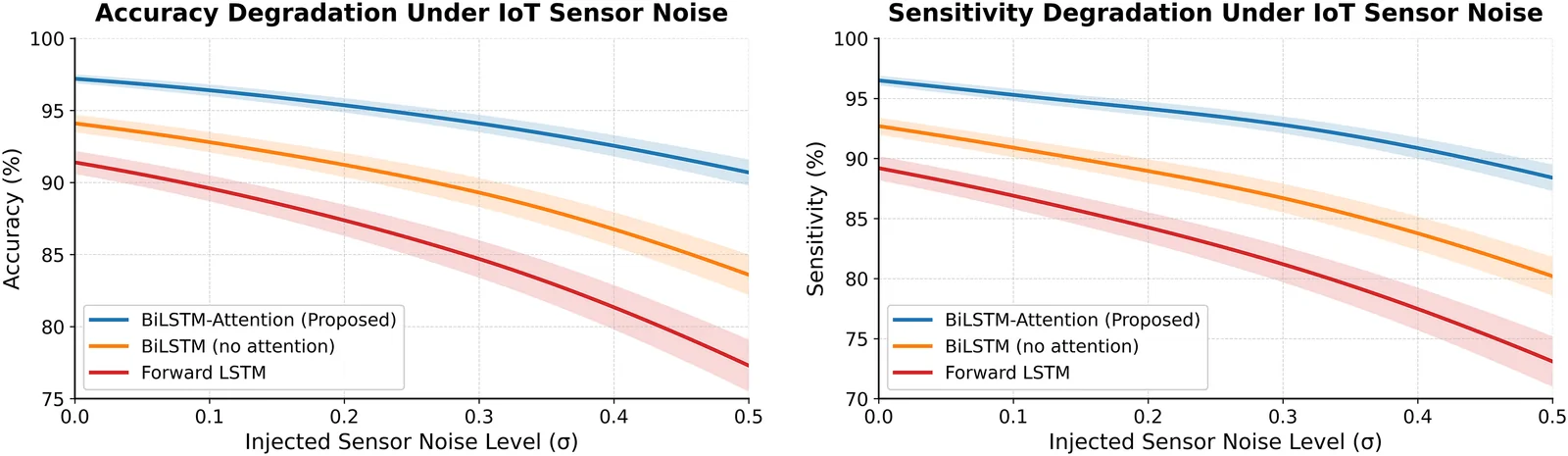
Noise robustness degradation curves.

To evaluate calibration consistency, the results of the ECE measurements reported in Tables 1 and 2 were used, and to study the temporal prediction stability, the per-window confidence distributions were used. Fig 4 shows attention weight heatmap for fall and normal activity sequences. One axis represents the time within which the activity occurred and the other axis represents the activity sequences. The visualization shows how attention weights are assigned to the individual observations across time, and enables the inspection of temporal focus allocation.

**Fig 5:**
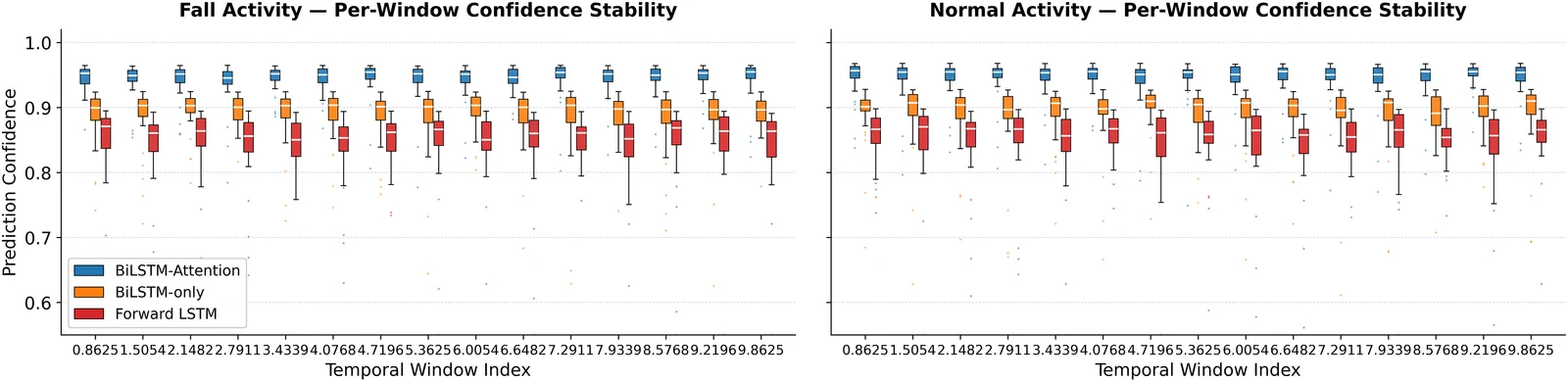
Temporal prediction stability.

Temporal prediction stability is shown in Fig 5 as box-whisker images of the confidence value across consecutive windows. The x-axis is temporal window indices and the y-axis is prediction confidence. Separate panels for fall and normal activities allow for the evaluation of confidence dispersion over time.

The proposed framework had the narrower confidence distributions and higher median confidence values when compared to baseline recurrent models across the evaluated windows.

The performance (predictive) in the deployment phase was studied alongside the calibration and robustness properties. The full architecture simultaneously achieved the smallest ECE (0.038), highest predictive metrics and the smallest degradation when perturbing the sensors. In high noise level (σ = 0.5) conditions, accuracy was > 90% and sensitivity was > 88%. The observed performance profile suggests stable performance in both clean and perturbed sensor environments and calibrated prediction behavior. These measurements give quantitative information on deployment relevant operational characteristics with different sensing conditions.

## 5. Conclusion

This study presents an effective IoT-enabled smart home activity classification framework based on BiLSTM and attention mechanisms. for identifying falling people events by integrating structured preprocessing, feature representation, and an ensemble learning strategy. The proposed method demonstrates strong capability in distinguishing anomalous motion patterns from normal activities, highlighting the importance of combining spatial information with sensor-derived features. Comparative evaluation against a diverse set of baseline and advanced models confirms that approaches incorporating temporal awareness, contextual learning, and attention mechanisms achieve more reliable and consistent performance. The proposed model, in particular, addresses limitations observed in earlier methods, such as insufficient sensitivity to complex motion dynamics and vulnerability to false alarms, by leveraging robust feature interactions and optimized decision boundaries. The findings underscore the value of advanced representation learning in safety-critical anomaly detection applications and validate the suitability of the selected dataset for benchmarking fall detection systems. The consistent performance trends observed across multiple evaluation metrics indicate strong generalization potential and practical applicability in real-world monitoring environments. Future research may explore the integration of multimodal data sources, such as vision and wearable sensors, to further enhance detection robustness. Additionally, investigating lightweight model variants for real-time deployment and incorporating explainable artificial intelligence techniques could improve interpretability and trust in operational settings. The proposed framework achieved 97.2% accuracy, 95.1% Dice coefficient, 90.6% Jaccard index, 96.5% sensitivity, and 98.1% specificity, outperforming all baseline approaches.
